# Colchicine-Induced Tetraploidy in Protocorms of *Aerides rosea* Lodd. ex Lindl. and Paxton. and Its Identification

**DOI:** 10.3390/plants13243535

**Published:** 2024-12-18

**Authors:** Li Wang, Pengrui Zheng, Hong Ge, Xin Zhao, Yaping Kou, Shuhua Yang, Xiaonan Yu, Ruidong Jia

**Affiliations:** 1 State Key Laboratory of Vegetable Biobreeding, Institute of Vegetables and Flowers, Chinese Academy of Agricultural Sciences, Beijing 100081, China; lilili0126@163.com (L.W.); zhengpengrui326@163.com (P.Z.); gehong@caas.cn (H.G.); zhaoxin01@caas.cn (X.Z.); kouyaping@caas.cn (Y.K.); yangshuhua@caas.cn (S.Y.); 2 Key Laboratory of Biology and Genetic Improvement of Flower Crops (North China), Ministry of Agriculture and Rural Affairs, Beijing 100081, China; 3 School of Landscape Architecture, Beijing Forestry University, Beijing 100083, China

**Keywords:** *Aerides*, chromosome doubling, orchid breeding, polyploid

## Abstract

*Aerides rosea* (Orchidaceae) boasts high ornamental value due to its pleasant aroma, foxtail spike, and elegant floral morphology. Inducing *A. rosea* to become tetraploid enhances horticultural traits and facilitates fertile intergeneric hybrids through crosses with other market-available tetraploid species. The experimental design involved the application of colchicine at varying concentrations—0.05%, 0.1%, and 0.2%—to a solid medium. Exposure durations were 5, 10, and 15 days, with treatments conducted under sterile conditions on 6-week-old protocorms post-germination. Results indicated that the protocorms were sensitive to colchicine concentrations exceeding 0.05%, with high concentrations leading to a mortality rate exceeding 50%. Flow cytometry (FCM) with 4′,6-diamidino-2-phenylindole (DAPI) staining confirmed a doubling of chromosome numbers in tetraploid plants (2n = 4x = 76) compared to diploid controls (2n = 2x = 38). Induction efficiency was significantly influenced by colchicine concentration and treatment duration. A 10-day treatment with 0.2% colchicine yielded a 70.00% tetraploid induction rate; however, considering protocorm survival, a 5-day treatment with 0.05% colchicine was preferable, achieving a 63.55% survival rate and a 56.67% tetraploid induction rate. Tetraploid plants exhibited distinct morphological traits, such as a more compact growth habit, thicker leaves, and increased stem and root thickness. Leaf morphology changes included larger stomata with reduced density, denser spongy mesophyll, and more pronounced venation. Tetraploids also demonstrated a 1.94-fold increase in genome size compared to diploids. The tetraploid genotypes developed in this study hold significant potential for future *Aerides* breeding programs.

## 1. Introduction

Orchidaceae, with approximately 30,000 species, is one of the largest families of flowering plants, exhibiting tremendous floral diversity [1]. Orchids are closely associated with forests, as many species primarily grow within forest ecosystems, where they play essential roles [2]. For hundreds of years, orchids have been prized by collectors and adventurers. Some of them, such as *Phalaenopsis* and *Cattleya*, have been cultivated as major ornamental crops. However, many species with significant horticultural value remain underutilized. *Aerides rosea*, among the vast array of orchid species, stands out as a particularly noteworthy example of the family’s diversity and ecological significance. This species has not been fully exploited for its horticultural potential. This rare and endangered [3] epiphytic orchid (living on trunks or branches of the host trees without absorbing nutrients and water from the hosts) is primarily distributed in the forests of southwestern China, Bhutan, northeastern India, Myanmar, Laos, and Vietnam [4]. Characterized by monopodial branching and succulent leaves, *A. rosea* produces long inflorescences densely packed with numerous flowers. The flowers are pink, pleasantly fragrant, and uniquely shaped, making this species highly valuable as an ornamental plant for both potted and cut flower markets [5]. As a member of the subtribe Aeridinae, *A. rosea* can hybridize with species from related genera within the subtribe. Its fragrant flowers make it a valuable resource for the production of many scented artificial hybrids and cultivars [6]. Within the subtribe Aeridinae, *Phalaenopsis* and *Vanda* are among the most popular and sought-after orchids in the global market [7]. According to the RHS database [8], there are 469 hybrid offspring involving *Aerides*, with 286 intergeneric hybrids with *Vanda* and 16 with *Phalaenopsis*. This indicates the extensive use of *Aerides* in intergeneric hybridization.

However, achieving successful hybridization can be challenging. Interspecific hybrids often suffer from reduced fertility due to problems with chromosome pairing and segregation during meiosis [9]. Polyploid breeding can address this issue of hybrid infertility by restoring fertility. Additionally, it is crucial to overcome barriers to hybridization. In some cases, obtaining desirable crosses is challenging due to differences in ploidy levels between potential parents. By adjusting the ploidy levels to be compatible before hybridization, these interploidy barriers can be overcome, allowing successful hybridization. [10]. Polyploids are defined as individuals with three or more sets of chromosomes in their somatic cells [11]. They are valuable to breeders due to their potential traits, including increased leaf thickness and length, larger and more textured flowers, prolonged blooming periods, thicker stems, greater biomass, and improved stress resistance compared to their diploid counterparts [12,13]. Polyploidy has been an important factor in the development of improved commercial varieties and hybrids of orchids [14]. However, there are currently no reports of tetraploid germplasm of *Aerides* being used for breeding.

Polyploid induction is an important method for genetic improvement and hybridization in orchids [15]. The primary mechanisms of natural polyploidy in orchids include endopolyploidy and hybridization [16]. Artificial induction is mainly achieved by applying antimitotic agents to tissues, organs, or whole plants [17]. Studies have shown that the efficiency of artificial polyploid induction in Orchidaceae depends on concentration, exposure time of the antimitotic agent, the type and age of explants, and the in vitro induction protocol [18]. The most widely used antimitotic agent for polyploid induction in plants is colchicine [19,20]. Significant progress has been made in inducing polyploid orchids in vitro using colchicine. This progress includes the successful somatic chromosome doubling in species such as *Phalaenopsis* [21], *Vanda* [22], *Cattleya* [23], *Cymbidium* [24], and *Dendrobium* [25], achieved through the application of colchicine in both solid and liquid media at concentrations ranging from 0.005% to 0.5%. It inhibits the formation of spindle fiber and effectively arrests mitosis at the anaphase stage. At this point, the chromosomes are doubled after cells pass another cycle. Orchid polyploidy can be induced either by culturing explants on a medium with colchicine at a precise concentration [21] or by submerging them in a colchicine solution [26]. Nonetheless, there are no reports on the polyploidization of *A. rosea*.

In addition to an effective system for polyploidy induction, confirmation methods are crucial for obtaining polyploids. Flow cytometry (FCM) is a rapid and relatively accurate method for the large-scale identification of artificially induced polyploids [27]. Chromosome counting in mitotic cells of root tips is an accurate procedure to determine ploidy, but it is time consuming and requires significant expertise. Therefore, attempts have been made to find indirect methods for ploidy determination. The area of stomata has been used to differentiate diploid and tetraploid regenerants of orchids [15]. The number of chloroplasts per guard cell pair [28] and differences in stomata density [29] have also been used as criteria in the distinction between diploid and tetraploid plants.

Inducing tetraploidy in *A. rosea* not only improves its horticultural traits but also enables crosses with other market-available tetraploid species, resulting in fertile intergeneric hybrids. In this study, we achieved tetraploids in *A. rosea* through in vitro colchicine treatment of protocorms. The resultant polyploid plants were identified based on morphological characteristics and confirmed via FCM and chromosome counts in root tip mitotic cells.

## 2. Materials and Methods

### 2.1. Plant Material and In Vitro Protocorm Establishment

**Figure 1:**
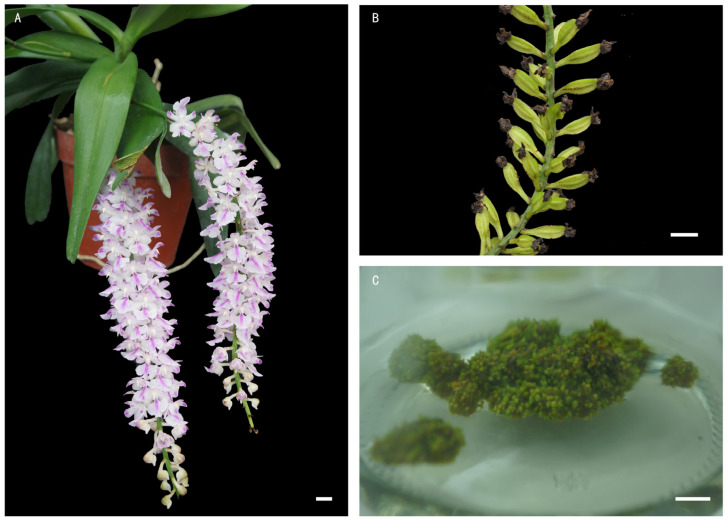
Morphology of *A. rosea* flowers, pods, and protocorms. (**A**) Flowers; (**B**) fruit pods; and (**C**) 6-week-old protocorms. Bars = 1 cm.

*A. rosea* plants (2n = 2x = 38) were cultivated in the greenhouse of the Institute of Vegetables and Flowers, Chinese Academy of Agricultural Sciences, located in Beijing, China. The plants were hand pollinated at full bloom in early June 2021, and capsules were harvested before dehiscence in late September. Detailed phenotypic characteristics are shown in Figure 1.

To produce in vitro protocorms, the capsules were disinfected in 75% ethanol for 30 s, washed once with sterile water, then were surface disinfected in a 10% (*v*/*v*) NaClO solution with an active chlorine content of 6–14% for 15 min. After rinsing three times with sterile distilled water, the capsules were cut with a sterile blade. Using sterilized tweezers, the capsule was held at one end above the mouth of the culture bottle, and carefully, the seeds were scraped out from the capsule into the culture bottle using the tip of the blade. The seeds were then spread onto the surface of the 1/2 Murashige and Skoog (MS) culture medium [30] with 5 g·L^−1^ carrageenan [31], 20 g·L^−1^ sucrose, and 100 mL·L^−1^ coconut water [32] at pH 5.8. The cultures were incubated at 25 °C under a 16/8 h light/dark photoperiod at a light intensity of 40 μmol/m²/s provided by white florescent tubes. Protocorms developed 6 weeks after sowing. Subsequently, protocorms with shoot apical meristem were used as explants for polyploid induction (Figure 1C).

### 2.2. Polyploidy Induction

Six-week-old protocorms of *A. rosea* were used for colchicine treatment. The protocorms used in the study were approximately 1–2 mm in diameter. They were cultured in the 1/2 MS culture medium with 1.0 mg·L^−1^ 6-benzylaminopurine, 5 g·L^−1^ carrageenan, 20 g·L^−1^ sucrose, 1.5 g·L^−1^ activated carbon, and 100 mL·L^−1^ coconut water, supplemented with colchicine at concentrations of 0, 0.05%, 0.1%, and 0.2% for 5, 10, and 15 days (Table 1). The colchicine solutions were sterilized by passing them through a 0.45 µm Millipore filter [21]. The experiment followed a completely randomized design with a factorial arrangement. Fifteen protocorms were used per replication, and three replicates were established for each colchicine treatment. After exposure to colchicine, the protocorms were rinsed three times using sterile distilled water and transferred onto fresh antimitotic-free seedling formation medium (growth conditions as mentioned above). The surviving protocorms were subcultured in medium and developed into plantlets within two months. Survival rates of protocorms were recorded at 6 weeks after colchicine treatment. The surviving protocorms were then transferred to a similar medium for further ploidy level analysis.

### 2.3. Flow Cytometry Polyploidy Analysis

Fresh leaves of 7-month-old seedlings were harvested after colchicine treatments for ploidy level identification using a CyFlow ploidy analyzer (Sysmex-Partec GmbH, Norderstedt, Germany). Sample preparation was performed according to the CyStain UV Precise P kit’s (Sysmex-Partec GmbH, Norderstedt, Germany) manual with minor modifications as follows: Samples were chopped in 400 µL nuclear extraction buffer for 30 s using a razor blade. The nuclei were mixed with 1600 µL of 4′,6-diamidino-2-phenylindole (DAPI) staining buffer and filtered through a 30 µm Cell-Trics filter (Sysmex-Partec GmbH, Norderstedt, Germany) into a sample tube. For each experimental setup, samples of young leaves from the in vitro seedlings of untreated (control) diploid *A. rosea* were selected as the calibration standard to adjust the gain of the flow cytometer such that the first G0/G1 peaks were set at channel 100 on a 1000 channel scale. Histograms were generated after analyzing ≥5000 nuclei using the De Novo FCS Express 7 software (De Novo Software, Los Angeles, CA, USA). The number of polyploid plants was recorded based on FCM analysis.

### 2.4. Morphological Analysis

Fifteen diploid and fifteen tetraploid plantlets were cultured in a plant tissue culture laboratory for 24 months. Morphological characteristics, including plant height, stem diameter, leaf count, maximum leaf length and width, leaf thickness, root count, and maximum root diameter, were analyzed. Leaf and root counts were directly recorded, while other parameters were measured with a digital vernier caliper.

### 2.5. Stomatal Observation and Leaf Anatomy

Leaves from diploid and colchicine-treated tetraploid plants were selected. Three leaves from each plant were cut from the middle regions of the third fully expanded leaves from the top of the stem. To observe stomata characteristics, upper and lower epidermal peels were taken from the leaves using forceps. Each peel was mounted on a glass slide with a drop of water and then covered with a cover slip [33]. The slides were observed under a microscope (OLYMPUS BX53, Olympus Corporation, Tokyo, Japan). The stomatal length and width of 10 randomly selected stomata from each leaf were measured. Then, stomatal density was recorded in two randomly selected microscopic fields of view on the adaxial and abaxial sides of each leaf.

Diploid and tetraploid leaf samples (5 mm × 5 mm) were cut from the middle regions of the third fully expanded leaves from the top of the stem and fixed in FAA (5% formaldehyde: 5% acetic acid: 90% ethanol, *v*/*v*) at room temperature overnight. Dehydration was performed in a series of ethanol solutions (50%, 70%, 85%, 95%, 100%, 100% *v*/*v*) for 15 min each. The samples were then processed through a 1:1 xylene: ethanol (*v*/*v*) mixture, followed by two rounds of xylene clearing for 15 min each. Gradual infiltration with paraffin wax at 62 °C was performed, involving a 1:1 xylene: paraffin (*v*/*v*) mixture and three rounds of paraffin immersion for 4 h each. The samples were cut into 10 µm transverse sections using a semi-motorized rotary microtome (Leica RM 2255, Leica Biosystems, Nussloch, Germany) and stained with toluidine blue. Sections were observed under an optical microscope (OLYMPUS BX53, Olympus Corporation, Tokyo, Japan). Four samples were analyzed for each ploidy level. For each sample, at least three random cross sections were chosen to measure leaf, mesophyll, vein, and upper and lower epidermal thicknesses.

### 2.6. Chromosome Counting

Root tips approximately 0.5 cm in length were collected and pretreated with a 0.1% colchicine solution for 6 h at 20 °C in the dark to induce chromosome contraction. Following this, the root tips were fixed overnight in Carnoy’s solution (ethanol:glacial acetic acid = 3:1). The fixed samples were then washed twice with distilled water and dehydrated in a series of ethanol solutions of varying concentrations, ultimately being preserved in 75% ethanol at 4C. Cell wall separation was facilitated by immersing the root tips in 0.075 mol/L KCl for 30–60 min at 37 °C. Subsequently, the root tips were treated with 1 mol/L HCl and heated in a water bath at 60 °C for 10 min. The samples were then squashed in a modified phenol fuchsin solution and observed under a microscope (OLYMPUS BX53, Olympus Corporation, Tokyo, Japan) to determine the chromosome number.

### 2.7. Genome Size Measurement

The nuclear DNA content of three plantlets from each solid diploid and tetraploid were estimated using a CyFlow ploidy analyzer with a 532 nm laser light source. Due to the unknown genome size of diploid *A. rosea* and the extensive endopolyploidy in orchids, making them potentially inaccurate as reference standards [34], *Zea mays* L. ‘B73’ [35] was chosen as the internal reference standard. Fresh leaves from the samples and the internal reference standard of *Z mays* ‘B73’ were simultaneously chopped with a sharp razor blade according to the manual for the Partec Cystain Propidium Iodide (PI) Absolute P kit (Sysmex-Partec GmbH, Norderstedt, Germany). Histograms were generated after analyzing ≥2000 nuclei [36], and representative flow histograms were analyzed using the De Novo FCS Express 7 software.

Values for the nuclear DNA content of the test sample were estimated using the following formula [37]:

Sample 2C DNA content = [(sample G1 peak mean)/(standard G1 peak mean)] × standard 2C DNA content (pg DNA)

1 pg DNA = 0.978 × 109 bp

### 2.8. Statistical Analysis

Plant height was measured as the vertical distance from the base of the plant to the highest point of the plant; shoot diameter was measured across the root to the nearest millimeter scale; root length was measured from the leaf base to the tip of the roots to the nearest millimeter scale; root diameter was measured across the root to the nearest millimeter scale; leaf thickness was measured as the distance between the two surfaces at the mid-point of the leaf; leaf length was measured from the leaf base to the tip to the nearest millimeter scale; leaf width was measured across the leaf to the nearest millimeter scale; the total number of roots of each plant was counted and recorded; and the total number of leaves of each plant was counted and recorded. The data analysis was conducted using Excel 2016 for basic analysis. One-way ANOVA and mean comparisons among treatments using the LSD method (at alpha = 0.05) were performed using SPSS 22. The experiment was biologically replicated three times. The values in the table represent the range of data changes (±) with respect to the standard deviation.

## 3. Results

### 3.1. The Impact of Colchicine Treatment on Survival and Induction Rates

The different concentrations of colchicine (0.05%, 0.1%, and 0.2%) and varying exposure times (5 days, 10 days, and 15 days) significantly affect the survival and mutation rates of protocorms. Data indicate that when colchicine concentration exceeds 0.05%, protocorm survival rate drops significantly. For instance, at 0.05% concentration, the survival rate is 63.55% after 5 days of treatment, but it decreases to 16.93% after 15 days. Similarly, at 0.2% concentration, the survival rate is 34.92% after 5 days and drops to 6.69% after 15 days. These results show that higher concentrations of colchicine have strong toxicity to protocorms, leading to high mortality rates. Additionally, high concentrations (0.2%) of colchicine result in mortality rates exceeding 50% across all exposure times, with a mortality rate of 93.31% after 15 days of treatment (Table 1).

The interaction between colchicine concentration and exposure duration was crucial for the induction rate, highlighting the importance of optimizing both factors for in vitro tetraploidy induction in *A. rosea*. The highest survival rate, 70.00%, occurred in the 0.2% treatment for 10 days, although the actual survival rate was 29.74%. Compared to Treatment 8, which had the highest induction rate, Treatment 7 used the same concentration but had a shorter 5-day treatment duration. This led to a 6.33% decrease in tetraploid induction rate and a 5.18% drop in protocorm survival rate. Treatment 1, with a 0.05% concentration for 5 days, experienced a 13.33% reduction in induction rate but a 33.81% increase in protocorm survival rate.

**Figure 2:**
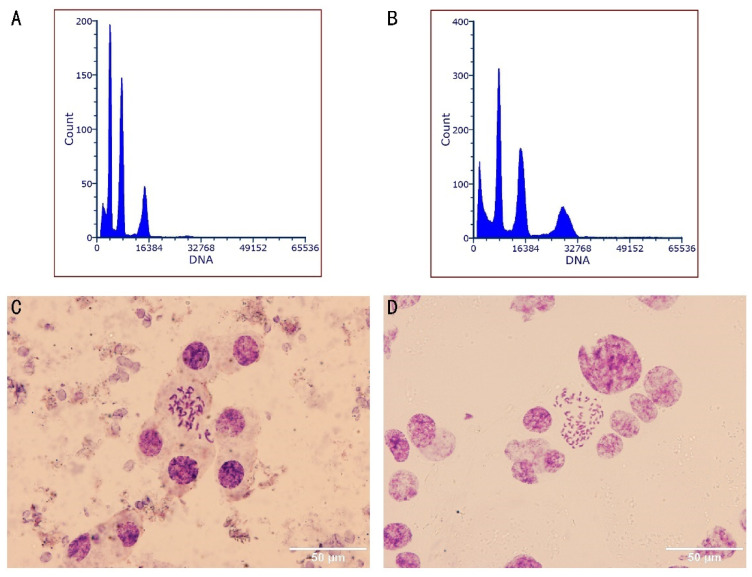
Ploidy detection of diploid and tetraploid plantlets via FCM and chromosomal counting. FCM histograms of DAPI-stained nuclei from in vitro leaf tissue for diploid (**A**) and tetraploid plantlets (**B**); chromosome of root tip cells from diploid 2n = 2x = 38 (**C**); and tetraploid 2n = 4x = 76 (**D**). Bars = 50 μm.

Tetraploid plants were identified using FCM on 121 surviving regenerated plants (Table 1). Diploid controls showed an initial G1 peak at channel 100 (Figure 2A), whereas a peak at channel 200 indicated tetraploidy (Figure 2B). All control plants were confirmed as diploid. Histograms in Figure 2 revealed three distinct peaks for both diploid and tetraploid samples, suggesting endopolyploidy in in vitro-regenerated cells. Chromosome counts confirmed the FCM results: diploids had 2n = 2x = 38 (Figure 2C), and tetraploids had 2n = 4x = 76 (Figure 2D).

### 3.2. Morphological Comparison

**Figure 3:**
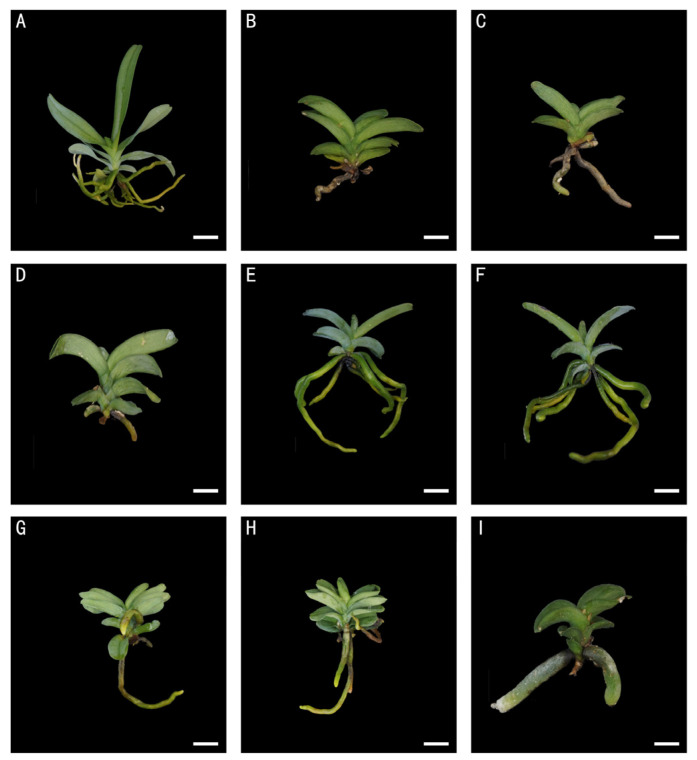
Morphologies of one-year-old diploid and tetraploid plantlets after colchicine treatment. (**A**) Diploid control plantlet; (**B**) 0.05% colchicine; (**C**) 0.05% colchicine; (**D**) 0.05% colchicine; (**E**) 0.05% colchicine; (**F**) 0.05% colchicine; (**G**) 0.1% colchicine; (**H**) 0.1% colchicine; and (**I**) 0.2% colchicine. Bars = 1 cm.

Twenty-four months after colchicine treatment, tetraploid plants (Figure 3B–I) exhibited significant morphological differences compared to diploid plants (Figure 3A). These differences included shorter stature, more compact growth, thicker stems and roots, rougher leaf texture, and thicker leaves. Under the same in vitro conditions, significant morphological differences were observed between diploid and tetraploid *A. rosea* plants (Table 2).

The average leaf width was 6.21 mm for diploids and 8.34 mm for tetraploids. Leaf thickness increased from 0.97 mm in diploids to 1.42 mm in tetraploids. Tetraploid plants had larger root diameters, measuring at 4.30 mm compared to 2.29 mm in diploids. Stem diameters also increased, from 2.01 mm in diploids to 3.22 mm in tetraploids. However, tetraploid plants were shorter and had shorter leaves than diploids. There was no significant difference in the number of leaves and roots between the two ploidy levels.

### 3.3. Stomatal and Mesophyll Cell Differences

**Figure 4:**
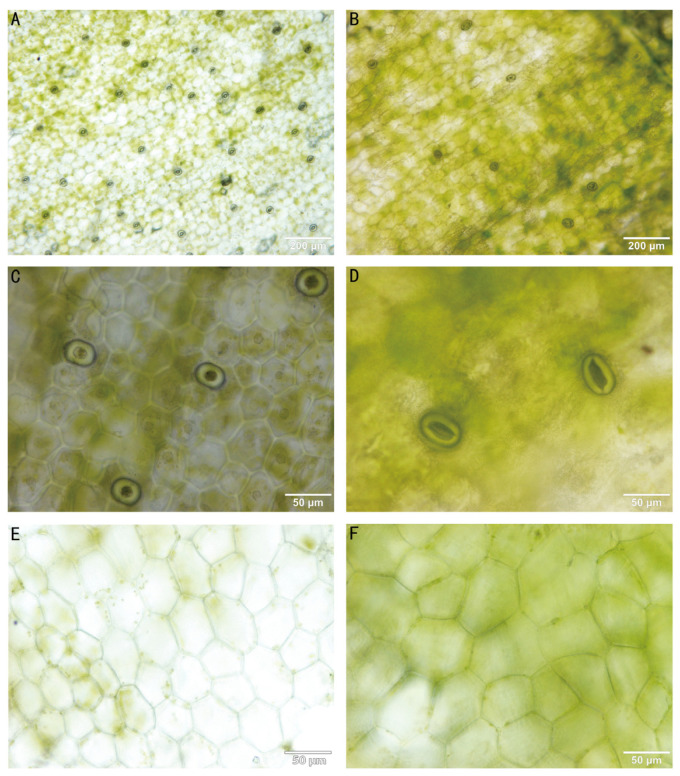
Leaf epidermal characteristics of diploids (**left**) and tetraploids (**right**). Abaxial epidermal stomatal density (**A**,**B**); stomatal size (**C**,**D**); and adaxial epidermal cell densities (**E**,**F**).

Differences in stomata between tetraploid and diploid plants provide a straightforward method for identifying polyploids. The stomatal density in tetraploids (Figure 4B) is significantly lower than in diploids (Figure 4A), with diploids at 31.09 µm and tetraploids at 8.06 µm. Tetraploid stomata (Figure 4D) are larger, with lengths and widths 39.51% and 52.86% greater than those of diploid stomata (Figure 4C) (Table 3). Both diploid (Figure 4E) and tetraploid (Figure 4F) plants have polygonal epidermal cells.

**Figure 5:**
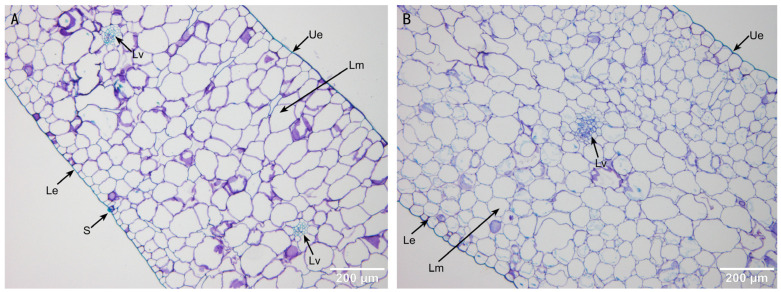
Leaf cross-section structure of diploids (**A**) and tetraploids (**B**). Ue—upper epidermis; Lm—leaf mesophyll; Lv—leaf vein; Le—lower epidermis; and S—stomata. Bars = 200 µm.

Longitudinal sections of the leaves show that both diploid (Figure 5A) and tetraploid (Figure 5B) orchid leaves have uniform mesophyll cells without distinct palisade or spongy tissues. The mesophyll cells are tightly packed and vary in shape from polygonal to elliptical. Stomata are distributed on the lower epidermis of the leaves. Table 3 shows that the thickness of epidermal cells, veins, and mesophyll in tetraploid leaves is significantly greater than that in diploid leaves.

### 3.4. Changes in DNA Content

**Figure 6:**
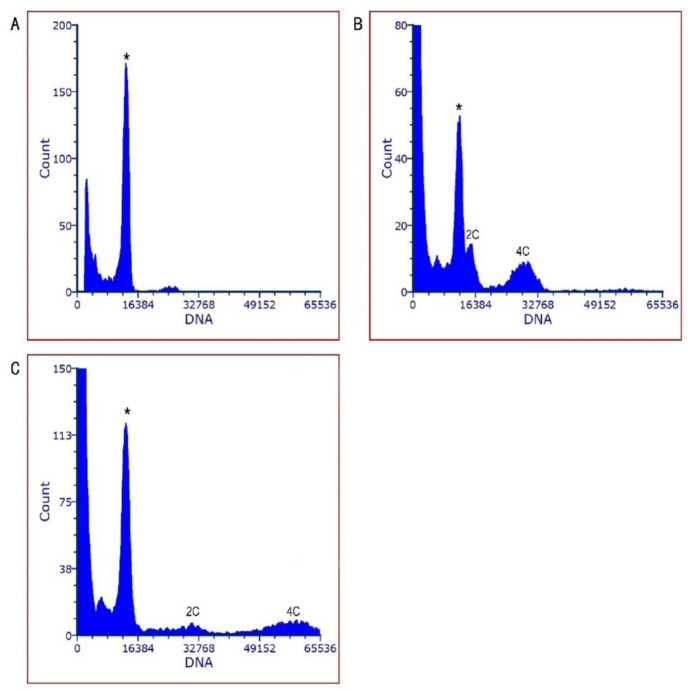
Genome size of *A. rosea* was estimated via FCM. DNA histograms of PI-stained nuclei of internal reference standard *Z. mays* ‘B73’, (**A**) the diploid, (**B**) and tetraploid (**C**). Peaks of the internal reference standard in the histograms of diploids and tetraploids were indicated by an asterisk. (* represents the 2C peak of *Z. mays* ‘B73’).

Histograms from the simultaneous analysis of nuclear suspensions from *A. rosea* and the internal reference standard (*Z. mays* ‘B73’) stained with PI displayed clear fluorescence peaks, confirming successful DNA content estimation. The mean CV values for both the reference and sample plantlets were below 5%. By utilizing *Z. mays* ‘B73’ as an internal standard (Figure 6A) in conjunction with diploids (Figure 6B) and tetraploids (Figure 6C), the average 2C DNA contents were found to be 2.90 and 5.62 Gb, respectively. The tetraploids exhibited a genome size that was 1.94 times larger than that of their diploid counterparts.

## 4. Discussion

The artificial induction of polyploids significantly increases the frequency of polyploid plants in breeding programs. An efficient in vitro mitotic chromosome doubling protocol depends on various factors, including species, explant type, antimitotic agents, exposure concentration and time, application method, and confirmation technique [19]. Protocorm-like bodies (PLBs) and protocorms are the primary explants for in vitro polyploid induction [18]. In orchids, thousands of seeds can be obtained from a single capsule, which after one or several months of in vitro germination, become numerous protocorms. Therefore, this study used the protocorms of *A. rosea* for induction. Research indicates that using protocorms for polyploid induction in orchids is widespread and highly effective. For instance, almost all reported *Dendrobium* species have used protocorms or PLBs as in vitro induction materials, with induction efficiencies reaching up to 80% [38].

The optimum concentration and duration of colchicine exposure differ among species, even within the same genus of plants. For instance, a 0.5% colchicine treatment for 10 days induced the most polyploids in *P. amabilis* Blume and *P. amboinensis* J.J.Sm. [39], while a 0.01% concentration for the same period was optimal for *P. pulcherrima* (Lindl.) J.J.Sm [40]. Moreover, the ideal colchicine concentration and exposure time for orchid polyploid induction depend significantly on the explant type and application method used. For *C. hybrid*, a 24 h soak of stem segments in a 0.05% colchicine solution resulted in a high induction rate of 28.2% [41]. However, when treating PLBs with the same concentration, it required 5 days to achieve its highest induction rate of 23.7% [42]. In our study, a colchicine concentration of 0.2% proved to be more effective in inducing polyploidy compared to concentrations of 0.05% and 0.1%. However, considering the balance between survival and induction rates, treatment with 0.05% colchicine for 5 days was also relatively effective. This protocol yielded an acceptable induction rate while maintaining a higher survival rate among the treated protocorms.

Colchicine has been shown to significantly reduce cell viability. In this experiment, our findings indicate that higher concentrations and longer exposure durations of colchicine absorbed by protocorms result in lower survival rates for both the protocorms and the resulting plantlets. This outcome is consistent with results in many plants, such as pear (*Pyrus communis* L.), mulberry (*Morus alba* L.), and perennial ryegrass (*Lolium perenne* L.) [43,44,45]. Low doses over extended periods are generally considered safer and more effective for inducing polyploidy without severely compromising plant health [46]. In this study, 0.05% colchicine reduced the survival rate of protocorms to below 50%, likely due to the greater sensitivity of *A. rosea* to colchicine [47]. Moreover, although low concentrations may not immediately affect cell viability, prolonged exposure can lead to cell death and reduced proliferation rates [48]. Additionally, negative effects of polyploidy can be observed during the growth stages. Manzoor et al. [49] observed that colchicine-treated gladiolus plants exhibited a range of leaf abnormalities, with the severity of these effects increasing at higher concentrations. In our study, the diploid leaves were upright and smooth, but the colchicine-treated leaves showed varying degrees of crimping and stunted growth. Specifically, leaves treated with 0.2% colchicine were semicircular and grew particularly slowly.

In polyploid induction, soaking plant tissues in colchicine tends to increase the occurrence of chimeras, which are plants with genetically different tissues. This is likely because the treatment does not uniformly affect all cells, leading to mixed ploidy levels within the same plant. Conversely, adding colchicine to solid culture media reduces chimera formation by promoting more stable polyploidy. The solid medium allows for more consistent exposure and treatment, thereby reducing variability. For instance, research on *D. wardianum* R. Warner has shown that cultivating protocorms in a culture medium with 75 μM colchicine for 30 days resulted in a 34% mutation rate and produced more stable polyploids compared to soaking. Using solid culture media helps to ensure uniformity in ploidy induction, thus reducing the formation of chimeras. [50]. Furthermore, higher concentrations of colchicine for shorter periods can cause severe malformations and reduce the production of tetraploid plants. Therefore, moderate concentrations with longer exposure periods have proven to be more effective and less toxic, reducing side effects and enhancing the overall induction rate of stable polyploids [51]. In this study, it is reasonable to increase the duration of colchicine exposure in the solid culture medium to 5–10 days.

The artificial induction of chromosome doubling in orchids, particularly through the use of colchicine, results in significant changes in stomatal and anatomical characteristics. Polyploid orchids typically exhibit larger stomata compared to their diploid counterparts. While the size of stomata increases, stomatal density often decreases in polyploids [50]. These changes in stomatal characteristics can influence photosynthetic efficiency and water regulation within the plant. Larger stomata can facilitate increased gas exchange, while reduced density might help minimize water loss under stressful conditions. Chromosome doubling leads to an overall increase in cell size, contributing to thicker leaves and stems [18]. Research indicates that colchicine treatment can lead to approximately a one-third reduction in plant height [9]. The extent of height reduction is influenced by the concentration of colchicine used, with higher concentrations exacerbating growth inhibition and reducing overall plant vigor [50]. Consequently, both plant morphology and stomatal features have been established as reliable indirect indicators for determining the ploidy level of *A. rosea*.

Moreover, endoreduplication, a phenomenon where cells have multiple sets of chromosomes, is prevalent in various orchid species [52]. In orchids, endoreduplication is linked to the rate of plant growth [53]. Tissue culture techniques that use endopolyploid cells, involving successive cycles of sectioning protocorms, have been adopted. For example, Chen observed endopolyploidy in diploid *P. aphrodite* Rchb. f. protocorms. Protocorms, measuring 3–5 mm in length after germination, were horizontally sectioned into equal parts before the expansion of the first leaf. The basal portion, with the sectioning surface facing upward, was placed into the culture medium for ex vitro regeneration. FCM determined that 34% of the PLBs in the first cycle of regeneration from the protocorms were polyploid [54]. In this study, endopolyploid cells were also detected in genotype-regenerated plantlets derived from diploid control protocorms. The ploidy level analysis showed that they were all diploids.

The genome size of artificially doubled plants does not always increase in direct multiples of the diploid genome. In our study, we used FCM with PI as the DNA fluorochrome on young leaf tissue to estimate the genome sizes of both solid diploids and colchicine-induced tetraploids. The tetraploids exhibited 1.94 times the DNA content of the diploids, with genome sizes of 2.90 and 5.62 Gb, respectively. These findings suggest that DNA loss or genome downsizing may occur after polyploid formation [55]. Furthermore, the extensive endoreduplication in orchid somatic tissues complicates genome size analysis. This challenge affects both whole and partial genome assessments, and the potential absence of 2C DNA content nuclei in the G1 phase could lead to the overestimation of genome size values. To address this, it is essential to randomly select multiple plants for analysis, with each plant being analyzed multiple times. Repeated measurements from the same plant can detect process variations and estimate differences between plants. While traditionally used leaves might yield inaccurate C-values, especially in species with gradual partial endoreduplication, young ovaries and pollinated pollen consistently show clear 2C and 1C peaks for G1 phase nuclei, making them optimal for orchid genome size studies, followed by leaf tips [56]. In this study, since the induced polyploid plants did not flower, leaf tips were used to measure genome size.

## 5. Conclusions

In this study, we report for the first time the induction of autotetraploids in *A. rosea* by treating diploid protocorm explants with colchicine in vitro. The appropriate concentration of colchicine and exposure time are crucial for increasing the rate of tetraploid induction. Based on our findings, the most effective approach, considering the balance between survival and induction rates, was treatment with 0.05% colchicine for 5 days. Compared to diploids, the tetraploids exhibited significant changes not only in morphological and anatomical characteristics but also in genome size. These tetraploid plants have the potential to serve as progenitors in the creation of novel fragrant germplasms, offering a diverse array of fragrances within the subtribe Aeridinae breeding programs.

## Figures and Tables

**Table 1:** Effects of colchicine on the survival rate and the mutation rate of *A. rosea* protocorms.

Antimitotic Agent	Treatment	Exposure Time (d)	Concentration %	Survival Rate %	Rate of Mutation %
Control	CK	0	0	98.56 ± 3.02 a	0
Colchicine	1	5	0.05	63.55 ± 6.08 b	56.67 ± 2.89 c
	2	10	0.05	47.61 ± 2.09 c	41.67 ± 2.89 e
	3	15	0.05	16.93 ± 1.7 f	41.67 ± 5.77 e
	4	5	0.1	46.09 ± 0.61 c	43.33 ± 2.89 e
	5	10	0.1	33.06 ± 1.69 de	46.67 ± 2.89 de
	6	15	0.1	10.24 ± 1.04 g	51.67 ± 2.89 cd
	7	5	0.2	34.92 ± 1.37 d	63.33 ± 2.89 b
	8	10	0.2	29.74 ± 1.37 c	70.00 ± 5.00 a
	9	15	0.2	6.69 ± 0.45 g	51.67 ± 2.89 cd

Note: Number of treated protocorms = 45. Data are the means ± SE of three replicates. Different lowercase letters in the same column indicate significant differences (*p* < 0.05).

**Table 2:** Comparison of morphological indexes of diploids and tetraploids induced by colchicine.

Phenotypic Characteristics	Diploid	Tetraploid
Plant height (mm)	49.77 ± 1.85 a	29.75 ± 1.04 b
Stem diameter (mm)	2.01 ± 0.03 b	3.22 ± 0.02 a
Number of leaves per plant	5.25 ± 0.5 a	4.25 ± 0.96 a
Leaf length (mm)	21.03 ± 1.04 a	11.13 ± 0.34 b
Leaf width (mm)	6.21 ± 0.03 b	8.34 ± 0.02 a
Aspect ratio of leaves	3.77 ± 0.24 a	1.70 ± 0.07 b
Leaf thickness (mm)	0.97 ± 0.14 b	1.42 ± 0.13 a
Root length (mm)	43.21 ± 4.89 b	26.82 ± 2.29 a
Root diameter (mm)	2.29 ± 0.18 b	4.30 ± 0.36 a
Number of roots per plant	5.25 ± 0.50 a	4.25 ± 0.96 a

Note: Data are the means ± SE of three replicates. Different lowercase letters in the same column indicate significant differences (*p* < 0.05).

**Table 3:** Leaf anatomical structure of diploid and tetraploid plantlets.

Stomatal Properties	Diploid	Tetraploid
Stomatal width (µm)	32.90 ± 1.83 b	45.90 ± 4.52 a
Stomatal height (µm)	30.02 ± 0.84 b	45.89 ± 5.86 a
Stomatal aspect ratio	1.10 ± 0.06 a	1.02 ± 0.18 a
Stomatal density (mm^2^)	31.09 ± 1.35 a	8.06 ± 1.12 b
Leaf vein diameter (µm)	62.43 ± 7.80 b	91.47 ± 12.91 a
Upper epidermal thickness (µm)	43.29 ± 3.43 b	54.81 ± 3.56 a
Lower epidermal thickness (µm)	30.44 ± 0.90 b	47.76 ± 3.58 a
Mesophyll thickness (mm)	0.83 ± 0.01 b	1.04 ± 0.05 a

Note: Data are the means ± SE of three replicates. Different lowercase letters in the same column indicate significant differences (*p* < 0.05).

## Data Availability

All data are included in the main text.
